# Alzheimer’s disease-linked *ACE* variants increase ACE1 catalytic activity and production of angiotensin II

**DOI:** 10.1016/j.jbc.2026.111171

**Published:** 2026-01-20

**Authors:** Miranda A. Salvo, Leah K. Cuddy, Dmitry Prokopenko, Elyse Watkins, Rudolph E. Tanzi, Robert Vassar

**Affiliations:** 1 Ken and Ruth Davee Department of Neurology, Northwestern University Feinberg School of Medicine, Chicago, Illinois, USA; 2 Genetics and Aging Research Unit and McCance Center for Brain Health, Department of Neurology, Massachusetts General Hospital, Boston, Massachusetts, USA; 3 Mesulam Center for Cognitive Neurology and Alzheimer’s Disease, Northwestern University Feinberg School of Medicine, Chicago, Illinois, USA

**Keywords:** Alzheimer’s disease, amyloid-beta (Aβ), angiotensin II, renin angiotensin system, single-nucleotide polymorphism (SNP)

## Abstract

Angiotensin-converting enzyme (*ACE*) is a validated risk locus for developing late-onset Alzheimer’s disease (AD). Although ACE1 expression and enzyme activity correlate with AD diagnosis, the mechanism by which this occurs is unclear. As a cell membrane-bound and shed peptidase, ACE1 is most commonly known to produce angiotensin II (Ang II), which has been linked to AD pathogenesis but also has been shown to cleave toxic Aβ_42_ to Aβ_40_, further complicating its role in AD. Previous work from our group characterized a rare *ACE* coding variant discovered through whole-genome and whole-exome sequencing of late-onset AD families: *ACE* rs4980 (R1279Q mutation) increases neuronal ACE1 and subsequent signaling through the central renin-angiotensin system (RAS), inducing age-associated hippocampal neurodegeneration. In this work, we report on two additional *ACE* variants associated with increased risk for developing AD: rs3730043 (T916M) and rs142947404 (N1036K). These variants were selected to investigate their effect on ACE1 protein processing and function in SH-SY5Y stable cell lines. In these cell lines, ACE1 protein trafficking to the cell surface was unaltered. Interestingly, however, both T916M and N1036K mutant cell lines resulted in increased ACE1 catalytic activity. Consequently, both mutant cell lines produced elevated levels of Ang II, a known mediator of neurodegeneration. This study provides further evidence for the role of ACE1 in AD and warrants continued research on this topic.

Alzheimer’s disease (AD) is the most prevalent form of dementia, affecting more than 50 million people worldwide (1). While the underlying mechanisms of dysfunction have been well studied in familial AD, where patients present with dominant mutations in genes such as *APP*, *PSEN1*, and *PSEN2*, approximately 90% to 95% of diagnosed cases are sporadic or late-onset Alzheimer’s disease (LOAD) (2). Unlike familial AD, there are no single, high-penetrance mutations known to directly cause LOAD. However, crucial advances in human genetics, including genome-wide association studies and sequencing of large patient cohorts, have identified numerous genes that act as risk factors for disease (3, 4). Developing a granular understanding of how these genes and their associated protein products may contribute to, or even drive, AD pathology remains a critical need in the field. The pathways by which genetic risk factors alter cellular homeostasis, promote amyloid or tau pathology, or impair neuronal and glial function remain poorly defined (5). Unraveling the molecular mechanisms by which these risk genes act pathogenically will advance our ability to design and implement therapeutics that can modify or delay the course of disease.

Angiotensin-converting enzyme (*ACE*) is a validated risk locus for LOAD (3, 6, 7). ACE1 protein is a membrane-bound peptidyl dipeptidase which can be shed from the cell surface and is well-characterized as a crucial component of the renin-angiotensin system (RAS). ACE1 acts in the peripheral RAS to convert angiotensin (Ang) I to Ang II peptide which can bind to the Ang II type 1 receptor (AT1R), causing vascular remodeling and increasing blood pressure (8, 9). Overactivation of AT1R can lead to atherosclerosis and hypertension, a modifiable AD risk factor (10, 11). Apart from Ang II, ACE1 has many other known substrates such as bradykinin, N-acetyl-Ser-Asp-Lys-Pro (Ac-SDKP), and Substance P (12). Importantly, the RAS is also present in the central nervous system, however its local function is not fully understood (9, 13). ACE1 protein and enzyme activity is increased in the brains of individuals with AD compared to age-matched controls (14). The role of ACE1 in AD is complicated due partly to its ability to cleave amyloid-β_42_ (Aβ_42_) *in vitro* (15, 16, 17), however *in vivo* evidence is conflicting (18, 19, 20, 21). Conversely, retrospective studies have found an inverse correlation between treatment with antihypertensive therapeutics, such as ACE1 inhibitors (ACEis) and Ang receptor blockers (ARBs), and dementia (22, 23, 24, 25). Furthermore, clinical studies have shown that ARBs can slow cognitive decline and reduce AD hazard ratio in hypertensive non-demented adults (24, 25).

We previously discovered pioneering evidence implicating ACE1 and the RAS in AD pathology by investigating a rare *ACE* coding variant, rs4980 (R1279Q mutation) in a knock-in mouse model (ACE1^KI/KI^). ACE1^KI/KI^ mice developed age-associated hippocampal neurodegeneration due to an increase in neuronal ACE1, causing an increase in Ang II and AT1R activation (26). This study revealed a clear relationship between ACE1 and neurodegeneration *in vivo*, which was mitigated by treating ACE1^KI/KI^ mice with an ACEi or ARB. Importantly, while this previous study utilized a select *ACE* variant, there are numerous rare AD-associated *ACE* variants that lack clear characterization.

In this work, we discovered two additional rare *ACE* coding variants through whole-genome and whole-exome sequencing (WGS, WES, respectively) of LOAD families. We characterized these variants by expressing them in SH-SY5Y neuroblastoma cell lines and found that both ACE1 mutations increase membrane-bound ACE1 enzymatic activity and consequently Ang II production. Taken together, our findings provide additional evidence connecting ACE1 activity, Ang II, and the RAS with LOAD and warrant further investigation about their role in AD pathophysiology.

## Results

### Identification of rare *ACE* variants through WGS and WES

To identify rare coding variants in *ACE*, we analyzed a deep (>40X) WGS dataset generated from the National Institute of Mental Health (NIMH) AD Genetics Initiative Study Sample. The dataset consisted of 446 AD families with affected and unaffected siblings (Table S1). After performing functional annotation, we focused on variants that were predicted to have medium to high impact on the corresponding protein. Next, we selected two such variants with a potential functional consequence: rs3730043 (T916M) and rs142947404 (N1036K) (Table 1).

**Table 1:** *ACE* variant presence in family- and population-based analyses

*ACE* variant	Point mutation	Family-based analyses		Population-based analyses	
		NIMH families	ADSP WGS families	ADSP WES unrelated subjects	ADSP WGS unrelated subjects
rs3730043	T916M	11 families: 11/15 affected carriers; 7/10 affected non-carriers	3 families: 2/2 affected carriers and 1/1 affected non-carrier	*P* = 0.04 in African Americans	Slightly over-represented in affected subjects
rs142947404	N1036K	1 family: 3/5 affected carriers; 1/1 affected non-carrier	Not present	*P* = 0.05 in all populations combined	Not significant

Detailed genetic data for each *ACE* variant discovered from the NIMH AD Genetics Initiative Study Sample. Descriptive data from family-based NIA ADSP WGS and statistical analysis from population-based NIA ADSP analyses are also included. Number of families and distribution of affected individuals are listed. *p*-values listed under “ADSP WES unrelated subjects” are in regard to the association of *ACE* variant presence with affected status (based on logistic regression with covariates). More detail is provided in the Results. NIMH, National Institute of Mental Health; NIA ADSP, National Institute on Aging Alzheimer’s Disease Sequencing Project; WGS, whole genome sequencing; WES, whole exome sequencing; SD, standard deviation.

T916M was present in 11 nuclear families (Fig. S1*A*). Among these families, there were 11 affected heterozygous (HET) carriers (7 females and 4 males; 1 *APOE* ε3/ε3, 3 *APOE* ε4/ε4 and 7 *APOE* ε3/ε4), four unaffected HET carriers [3 females (ages 69, 71 and 90+) and 1 male (age 58); 1 *APOE* ε3/ε3 and 3 *APOE* ε3/ε4], seven affected non-carriers (6 females and 1 male; 2 *APOE* ε4/ε4 and 5 *APOE* ε3/ε4), and three unaffected non-carriers (1 female and 2 males; all *APOE* ε3/ε4). N1036K is present in five out of six members of one family, all of which are female (Fig. S1*B*). Three affected carriers had *APOE* ε3/ε4 genotype; the two unaffected carriers had *APOE* ε3/ε3 and ε3/ε4 genotypes. The single non-carrier was an affected subject. Next, to validate these *ACE* variants in additional cohorts, we gained access to the National Institute of Aging Alzheimer’s Disease Sequencing Project (NIA ADSP) whole-genome/whole-exome AD datasets (reported in Table S1). We found T916M but not N1036K in the NIA ADSP family-based subset. T916M was present in three HET carriers from three nuclear families (one unaffected female (74) with *APOE* ε3/ε4, one affected female (75) with *APOE* ε3/ε3 and 1 affected male (75) with *APOE* ε3/ε4) (Fig. S1*A*). In addition, we found that T916M was significantly associated with AD (*P* = 0.04) in an AD case-control analysis of unrelated subjects in the African American population of the NIA ADSP WES dataset. N1036K was nominally significant in all populations of the NIA ADSP WES dataset (Tables 1 and S2).

### ACE1 stable cell lines display unaffected protein degradation dynamics and survival phenotypes

**Figure 1:**
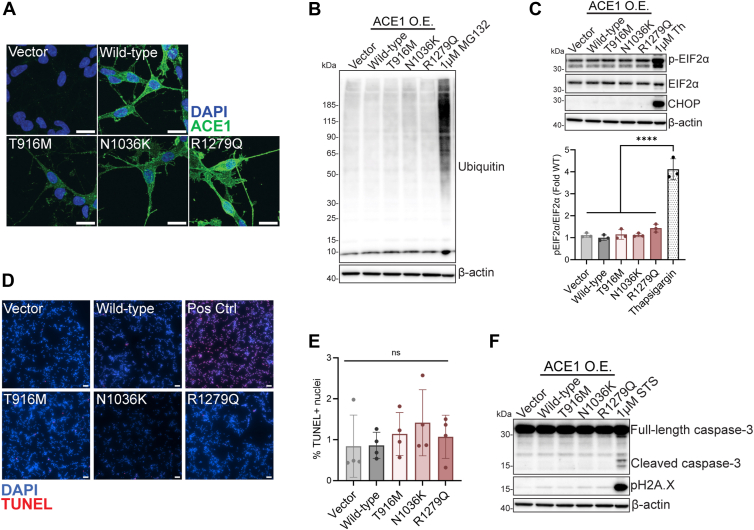
**ACE1 stable cell lines display unaffected protein degradation dynamics and survival phenotypes.***A*, expression of ACE1 protein in differentiated SH-SY5Y *ACE* stable cell lines. Scale bar 15 μm. *B*, Western blot showing proper clearance of ubiquitinated proteins in stable cell line whole cell lysate. 24-h treatment of 1 μM MG132 was used as a positive control for improper ubiquitin-proteasome system function. *C*, Western blot showing no increase in ER-associated stress. 24-h treatment of 1 μM thapsigargin (Th) was used as a positive control to induce ER-stress. pEIF2α normalized to EIF2α quantified below (one-way ANOVA with Tukey’s multiple comparisons test, *p* < 0.0001 Thapsigargin vs all other columns, ACE1 mutant cell line columns compared to each other were not significant). *D*, representative images of DAPI- and TUNEL-positive nuclei in stable cell lines. Scale bar 50 μm. One Vector coverglass was incubated with 1000 U/ml for 30 min to induce DNA double-stranded breaks as a positive control (Pos Ctrl). *E*, quantification of the percentage of TUNEL-positive nuclei per cell line. Results from 4 differentiations, 1 coverglass per experiment, points plotted as the average of three images per coverlgass for each differentiation (one-way ANOVA with Tukey’s multiple comparisons test, *p* > 0.05 all columns vs all columns). *F*, Western blot showing no signs of apoptosis or DNA double-stranded breaks in differentiated stable cell lines. 5-h treatment of 1 μM staurosporine (STS) was used as a positive control to induce apoptosis. O.E., overexpression.

Next, we used *in vitro* models to investigate the pathogenic roles of T916M and N1036K related to AD. ACE1 is predominately expressed in neurons in the hippocampus (27), therefore we chose to study the effect of selected *ACE* variants, as well as the previously characterized R1279Q mutation, in SH-SY5Y neuroblastoma cells. We generated SH-SY5Y cell lines which stably overexpress each *ACE* coding variant (T916M, N1036K, and R1279Q) or wild-type *ACE* (Wild-type). In addition, we created an SH-SY5Y cell line stably expressing the empty plasmid backbone as a control for *ACE* expression (Vector). We first confirmed that Wild-type and mutant ACE1 stable cell lines overexpress ACE1 protein compared to the Vector control line and display unaffected morphology (Fig. 1*A*). To determine whether overexpression of these *ACE* variants contribute to disruptions in protein homeostasis, we performed Western blot (WB) analyses on whole cell extract (WCE) and probed for known markers of proteostasis-related stress. There were no differences in the abundance of ubiquitinated proteins or endoplasmic reticulum stress when comparing the mutant lines to each other (Fig. 1, *B* and *C*). To next address if mutant ACE1 expression led to cell death, we performed TUNEL and WB analyses on fixed cells and WCEs, respectively. These findings confirmed that there were no differences in apoptosis, quantified by TUNEL staining and probing for active caspase-3 or H2A.X phosphorylation (Fig. 1, *D*–*F*). These findings led us to determine that ACE1 risk mutants do not result in AD pathology due to altered protein folding, degradation, or aberrant cell death.

### The R1279Q ACE1 mutant causes increased abundance of ACE1 protein on the cell surface

**Figure 2:**
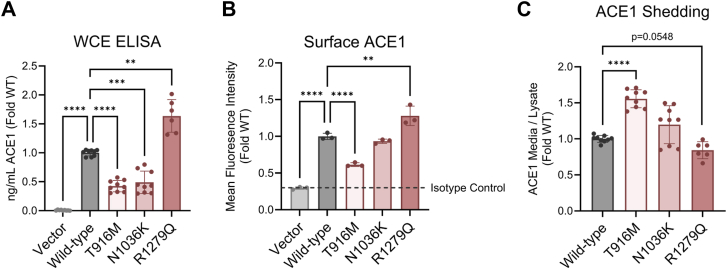
**The R1279Q ACE1 mutant causes increased abundance of ACE1 protein on the cell-surface.***A*, ACE1 protein measured in WCE *via* ELISA, three differentiations (two differentiations for R1279Q). Brown-Forsythe ANOVA test (*p* < 0.0001), Dunnett’s T3 multiple comparisons test Wild-type vs each mutant (vs Vector *p* < 0.0001, vs T916M *p* < 0.0001, vs N1036K *p* = 0.0001, vs R1279Q *p* = 0.0096). *B*, surface ACE1 protein measured by flow cytometry. Each data point represents the mean fluorescence intensity (MFI) of single cells from 20,000 cells counted per well, n = 3 wells per cell line. One representative experiment showed two separate differentiations. One-way ANOVA (*p* < 0.0001), Dunnett’s multiple comparisons test Wild-type vs each mutant (vs Vector *p* < 0.0001, vs T916M *p* < 0.0001, vs N1036K *p* = 0.5619, vs R1279Q *p* = 0.0013). *C*, ACE1 shedding assessed *via* ELISA measurement of ACE1 in conditioned media and normalized to ACE1 in WCE, three differentiations (two differentiations for R1279Q). Brown-Forsythe ANOVA test *p* < 0.0001), Dunnett’s T3 multiple comparisons test Wild-type versus each mutant (vs T916M *p* < 0.0001, vs N1036K *p* = 0.1427, vs R1279Q *p* = 0.0548).

ACE1 is predominately located on the plasma membrane where it can cleave substrates in the extracellular space, but it can also be shed from the plasma membrane and remain catalytically active in its soluble form (8). To investigate if the pathogenic mechanisms for ACE1 mutants are due to altered protein localization, we quantified ACE1 protein abundance in WCE *via* Enzyme-Linked Immunosorbent Assay (ELISA). We found that Wild-type had a significant increase in ACE1 protein compared to Vector, which expresses endogenous levels of ACE1. Additionally, T916M and N1036K had lower ACE1 protein compared to Wild-type, while R1279Q had higher ACE1 protein (Fig. 2*A*). Similar trends were also shown when quantifying ACE1 protein abundance through WB (Fig. S2, *A* and *B*). We also transiently transfected HEK293T cells with the same constructs used to create the SH-SY5Y stable cell lines and found no difference in ACE1 protein from WCE (Fig. S2, *C* and *D*). Therefore, ACE1 protein abundance may be attributed to individual stable cell line characteristics and not related to the ACE1 mutations. To test if the differences in protein abundance could be attributed to decreased ACE1 stability, we treated the stable cell lines with 50 μg/ml cycloheximide to halt protein translation and then measured ACE1 protein degradation in WCE over 32 h. We found that N1036K and R1279Q ACE1 mutants were cleared at similar rates to Wild-type while T916M had little clearance (Fig. S3, *A* and *B*), suggesting that it may increase the stability of ACE1. Based on these experiments, we can determine that ACE1 protein expression is stable and differences in WCE protein abundance are likely due to stable cell line characteristics.

To investigate ACE1 protein localization at the plasma membrane, we measured surface ACE1 abundance across our cell lines using flow cytometry. Compared to Wild-type, T916M had significantly reduced ACE1 surface abundance while R1279Q had significantly increased ACE1 surface abundance (Fig. 2*B*). To parallel this finding, we measured ACE1 shedding from the plasma membrane by quantifying the excreted ACE1 protein in the media normalized to ACE1 protein in WCE *via* ELISA. This analysis revealed that the T916M mutant cell lines have significantly increased ACE1 shedding (Fig. 2*C*), coinciding with the reduced surface ACE1 expression (Fig. 2*B*). R1279Q trends towards reduced ACE1 shedding, which is consistent with our previous report (26). Overall, these differences in plasma membrane-bound ACE1 and production of soluble ACE1 (sACE) are important when considering the effects of these mutations on substrate cleavage and local RAS signaling, particularly when other AT1R-expressing cell types are present to engage in downstream interactions.

### T916M and N1036K mutants have increased membrane-bound ACE1 catalytic activity

**Figure 3:**
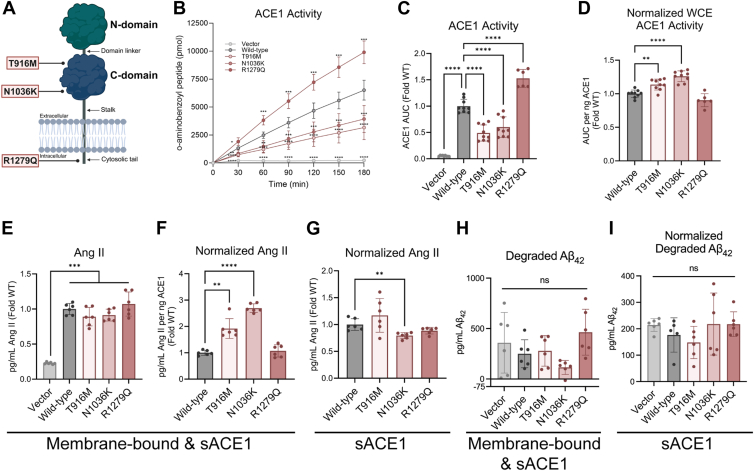
**T916M and N1036K mutants have increased membrane-bound ACE1 catalytic activity.***A*, figure chematic of membrane-bound ACE1. Created with BioRender. *B*, production of fluorescent o-aminobenzoyl peptide over 3 hours, three differentiations (2 differentiations for R1279Q) (two-way ANOVA, Dunnett’s multiple comparisons test, see Data Set 1 for details). *C*, ACE1 activity in WCE measured by area under the curve (AUC) of o-aminobenzoyl peptide over 3 hours. Plotted as fold of Wild-type (WT). Brown-Forsythe ANOVA test (*p* < 0.0001), Dunnett’s T3 multiple comparisons test Wild-type vs each cell line (vs Vector *p* < 0.0001, vs T916M *p* < 0.0001, vs N1036K *p* = 0.0006, vs R1279Q *p* = 0.0004). Captopril-treated sample average: 0.0465 AUC Fold WT. *D*, AUC normalized to WCE ACE1 protein per reaction (2 differentiations for R1279Q). Plotted as Fold WT. One-way ANOVA (*p* < 0.0001), Dunnett’s multiple comparisons test Wild-type vs each mutant (vs T916M *p* = 0.0024, vs N1036K *p* < 0.0001, vs R1279Q *p* = 0.0693). *E*, pg/ml of Ang II in stable cell line conditioned media after cells were treated with 500 nM Ang I for 1 h, plotted as fold of WT. Brown-Forsythe ANOVA test (*p* < 0.0001), Dunnett’s T3 multiple comparisons test all cell lines vs each other (Vector vs Wild-type *p* < 0.0001, Vector vs T916M *p* = 0.0005, Vector vs N1036K *p* < 0.0001, Vector vs R1279Q *p* = 0.0005, all other comparisons *p* > 0.05). Captopril-treated sample average: 0.0059 pg/ml Ang II Fold WT. *F*, pg/ml Ang II measured in conditioned media after 1 h treatment with 500 nM Ang I, normalized to WCE ACE1 protein per reaction, two differentiations. Plotted as Fold WT. Brown-Forsythe ANOVA test (*p* < 0.0001), Dunnett’s T3 multiple comparisons test Wild-type vs each mutant (vs T916M *p* = 0.0031, vs N1036K *p* < 0.0001, vs R1279Q *p* = 0.8669). *G*, pg/ml Ang II produced from incubating 3 ng sACE1 and 100 nM Ang I for 1 h *in vitro*, two differentiations. Plotted as Fold WT. Brown-Forsythe ANOVA test (*p* = 0.0242), Dunnett’s T3 multiple comparisons test Wild-type vs each mutant (vs T916M *p* = 0.4315, vs N1036K *p* = 0.0254, vs R1279Q *p* = 0.9927). Captopril-treated sample average: 0.0115 pg/ml Ang II Fold WT. H) pg/ml of degraded Aβ_42_ measured by ELISA in conditioned media after 24-h treatment with 1 nM Aβ_42_, two differentiations. Brown-Forsythe ANOVA test (*p* = 0.072), Dunnett’s multiple comparisons test Wild-type vs each mutant (vs Vector *p* = 0.8787, vs T916M *p* = 0.9936, vs N1036K *p* = 0.223, vs R1279Q *p* = 0.2669). Captopril-treated sample average: 178.76 pg/ml Aβ_42_. I) pg/ml of degraded Aβ_42_ measured by ELISA after incubating 3 ng sACE1 and 1 nM Aβ_42_ for 24 h *in vitro*, two differentiations. Plotted as Fold WT. Brown-Forsythe ANOVA test (*p* = 0.3583), Dunnett’s T3 multiple comparisons test Wild-type vs each mutant (vs Vector *p* = 0.6515, vs T916M *p* = 0.9068, vs N1036K *p* = 0.9067, vs R1279Q *p* = 0.6765). Captopril-treated sample average: 92.8 pg/ml Aβ_42_.

One documented role for ACE1 in neuronal cells is to enzymatically cleave substrates used in different cellular processes (8, 28, 29). ACE1 contains two catalytic domains that extend into the extracellular space (the N-domain and C-domain) (Fig. 3*A*). Although these domains are 60% homologous, they have different substrate specificities. For example, the C-domain predominately cleaves Ang I to Ang II, and the N-domain has been shown to predominately cleave Aβ_42_ to Aβ_40_ (8, 12, 15). Notably, both T916M and N1036K are located on the catalytic C-domain of ACE1 (Fig. 3*A*); therefore, we sought to address whether the mutations altered ACE1 catalytic activity. We first tested this in the WCE of ACE1 stable cell lines. Interestingly, all ACE1 mutants had differing levels of ACE1 activity compared to Wild-type when measuring the cleavage of an ACE1-specific fluorometric peptide over 3 hours in WCE (Fig. 3, *B* and *C*). Samples treated with an ACE1 inhibitor (captopril) confirmed the specificity of the fluorometric peptide (captopril-treated sample average: 0.0465 ACE1 activity Fold WT). To address the specific catalytic changes of mutant ACE1, we normalized the activity levels from this assay to ACE1 protein in WCE *via* ELISA, therefore providing a measurement of ACE1 activity per nanogram of ACE1 protein. This quantification revealed T916M and N1036K to have higher ACE1 catalytic activity compared to the other cell lines (Fig. 3*D*).

We next chose to measure membrane-bound and soluble ACE1 activity by treating differentiated stable cell lines with 500 nM Ang I for 1 h, then measuring the amount of Ang II, the ACE1 substrate cleavage product, in the conditioned media (CM). This resulted in a 4-fold increase in Ang II production in the ACE1 overexpressing cell lines compared to Vector (Fig. 3*E*). Samples treated with captopril confirmed that the Ang II produced by the cells was ACE1-specific (captopril-treated sample average: 0.0059 pg/ml Ang II Fold WT). When Ang II was normalized to WCE ACE1, as was performed in Figure 3*D*, this resulted in a significant increase in Ang II production from T916M and N1036K compared to Wild-type (Fig. 3*F*). Although this result is in line with our findings from the ACE1 activity assay (Fig. 3*D*), both membrane-bound and soluble ACE1 may be responsible for Ang II production in this current experiment. To remedy this, we performed this reaction in a cell-less model by combining sACE1 collected from stable cell line CM and Ang I. sACE1 was measured by ELISA first so that 3 ng of sACE1 was added to 100 nM Ang I for each reaction, resulting in Ang I cleavage by sACE1 alone. Interestingly, all ACE1 overexpressing cell lines except for N1036K produced similar amounts of Ang II (Fig. 3*G*), showing that membrane-bound mutant ACE1 is most responsible for cleaving Ang I to Ang II. N1036K sACE1 produced less Ang II compared to Wild-type, suggesting that its catalytic abilities diminish after being shed from the plasma membrane (Fig. 3*G*). These two assays show that T916M and N1036K alter the catalytic properties of membrane-bound ACE1, while R1279Q has increased ACE1 activity due to its increased protein abundance.

Finally, to address a relevant link between ACE1 and AD, we tested the ability of ACE1 to cleave Aβ_42_, which is predominately cleaved by the catalytic N-domain of ACE1. Previous work has shown that ACE1 can cleave Aβ_42_ to Aβ_40_ (15, 17), although some studies challenge the notion that ACE1 degrades Aβ_42_ at an appreciable amount *in vivo* (20, 26, 30). We treated differentiated stable cell lines with 1 nM Aβ_42_ for 24 h, then measured the amount of Aβ_42_ remaining in the CM *via* ELISA. The difference between the initial and remaining amount of Aβ_42_ was calculated. Surprisingly, there were no differences in the amount of Aβ_42_ between any cell line, including Vector (Fig. 3*H*), indicating that any Aβ_42_ degradation or loss was not solely dependent on ACE1. Samples treated with captopril also confirmed this conclusion (captopril-treated sample average: 178.76 pg/ml Aβ_42_). We could not detect Aβ_40_ in the CM of those samples *via* ELISA (data not shown). In this experiment, it is possible that Aβ_42_ may have stuck to the membrane, been phagocytosed by the cell, or been cleaved by other amyloid-degrading proteases expressed by the SH-SY5Y cells. Therefore, we also tested the amyloid-degrading ability of sACE1 in the same cell-free method as previously described. Again, there were no differences in the amount of degraded Aβ_42_ after 24 h between all cell lines. Captopril-treated samples reduced Aβ_42_ degradation slightly compared to the non-inhibited samples (captopril-treated sample average: 92.8 pg/ml Aβ_42_); however, it did not fully inhibit degradation, confirming that other amyloid-degrading proteases are dominant in this experimental setup (Fig. 3*I*). Based on these results, we conclude that ACE1 in our stable cell lines does not degrade Aβ_42_ at an appreciable amount.

## Discussion

**Figure 4:**
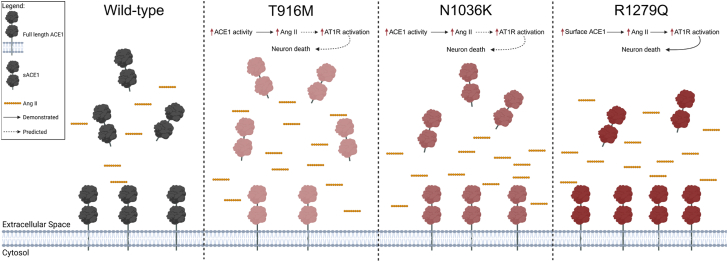
**Summary of primary findings and predicted effects in the central nervous system.** ACE1 is a membrane-bound dipeptidyl-peptidase but can be cleaved primarily by ADAM10 to release the two catalytic domains into the extracellular space (far *left panel*). Under normal conditions, membrane-bound ACE1 cleaves Ang I to form Ang II (*yellow* peptide icon) which can bind to receptors such as AT1R and activate intracellular signaling cascades. Ang II can also be further degraded into other angiotensin peptide products by proteases like ACE2 and aminopeptidase A. sACE1 can also cleave Ang I to Ang II, albeit at a lower rate than membrane-bound ACE1 depending on the microenvironment. Previous work has shown the AD-associated R1279Q mutation in knock-in mice increases ACE1 presence at the neuronal membrane which caused increased Ang II production and AT1R activation, leading to neuronal death (far *right panel*). We have demonstrated in this work that R1279Q stable cell lines have increased membrane-bound ACE1. Two additional AD-associated ACE1 mutations, T916M and N1036K, increase Ang II production through a different mechanism than R1279Q. These mutations increased the catalytic activity of membrane-bound ACE1 which led to an increase in Ang II (*middle left* and *middle right panels*). In the central nervous system, this increase in Ang II can over-activate AT1R and lead to neuron death, as was shown in the R1279Q ACE1^KI/KI^ mice. Notably, T916M had decreased ACE1 at the plasma membrane which was complimented by increased ACE1 shedding. This may contribute to our proposed mechanism of neuron death by increasing the amount of sACE1 available for Ang II production in the extracellular space, or soluble mutant ACE1 may play an additional role as a signaling molecule. Further work *in vivo* is required to understand the way in which these two mutations affect ACE1, RAS, and AD pathogenesis in the central nervous system. The schematic was created with BioRender.

Taken together, our data highlight two novel AD-associated *ACE* variants that alter the functional properties of ACE1. The T916M and N1036K mutations increase the production of Ang II, primarily through membrane-bound ACE1, in neuroblastoma stable cell lines (Fig. 4). Furthermore, we showed that these two mutations are processed and trafficked to the cell membrane, all while preserving protein degradation dynamics and survival phenotypes of the stable cell lines.

Our findings regarding the increase in ACE1 catalytic activity align with human data showing that AD patient brains have increased ACE1 activity compared to age-matched controls (14). Although neither the T916M nor N1036K mutations occur within the active site of the ACE1 catalytic C-domain, they both occur on helix-capping residues (31). T916M mutates the N-cap of α-helix 11 while N1036K mutates the C-cap of α-helix 14 in the C-domain. N- and C-capping residues are important for α-helical structure, although mutations in the former are more likely to cause α-helix instability than the latter (32, 33). Threonine is one of the preferred N-capping residues, while methionine is one of the non-preferred residues, suggesting that this may cause α-helix instability and conformational changes to the C-domain (33). By using AlphaFold3 to create a predictive structure of T916M, we found that the mutated residue altered the direction of the side chain (Fig. S4, *A*1 and *A*2). Furthermore, comparing the predictive T916M structure with the wild-type structure revealed alterations in the two areas of the domain that may increase access to the substrate pocket (Fig. S4, *D*1, *D*2 and *E*1, *E*2). When considering the N1036K mutation at a C-capping residue, both asparagine and lysine are preferred residues (33). We also did not observe large conformational differences between the N1036K predictive structure compared to wild-type (Fig. S4, *C*3, *D*3 and *E*3). However, the mutated lysine residue may alter ACE1 dimerization or binding to other proteins, thus influencing its activity, which has been reported and will be discussed further below (34, 35). Importantly, further analysis such as solving the crystal structure of mutant ACE1 or performing molecular dynamics simulations would be needed to conclude the effects of each mutation on any structural changes.

An integral factor of our experiments lies in the catalytic differences of membrane-bound and soluble ACE1. We showed that ACE1 catalytic activity is increased in T916M and N1036K mutants when assaying ACE1 activity directly in WCE and in its ability to cleave Ang I to Ang II. However, when sACE1 was collected and incubated with Ang I, we found that apart from N1036K (which produced lower levels of Ang II), all mutant forms of ACE1 produced similar amounts of Ang II to wild type. This is an important distinction which shows that membrane-bound ACE1 contributed more to Ang I cleavage than sACE1 in our model system. It has been reported that the catalytic activity of membrane-bound ACE1 is higher than that of sACE1, however both perform optimally in specific microenvironments depending on pH and ion availability (36). The excretion of sACE1 from the membrane was originally thought to influence local RAS signaling since it is catalytically active in both forms (8, 37). However, transgenic mice only expressing the secreted form of ACE1 could not maintain normal blood pressure even though it was sufficient for restoring kidney functions found in ACE1 null mice, suggesting that sACE1 may have other functions besides substrate cleavage (38) such as bradykinin receptor expression regulation in vascular smooth muscle cells (39). Furthermore, sACE1 can readily dimerize and affect N- and C-domain conformations which is dependent on glycosylation (40). The differences seen in membrane-bound and soluble ACE1 in T916M and N1036K may be due to dimerization, aggregation, loss of post-translational modifications, or sub-optimal catalytic microenvironment after being shed from the membrane.

We also showed that Aβ_42_ degradation is not primarily due to ACE1 in our stable cell lines. This conclusion is supported by two findings: first, the Vector cell line, which expresses approximately 100-times less ACE1 compared to the overexpressing Wild-type cell line, degraded the same amount of Aβ_42_ after 24 hrs, whether treated in live cells or in our cell-less method (Fig. 3); second, the ACE1 inhibitor captopril did not alter Aβ_42_ degradation after 24 hrs. Therefore, any reduction in Aβ_42_ was not ACE1-dependent, so we could not normalize the remaining Aβ_42_ levels to ACE1 protein in WCE as we had done previously with our Ang II measurements. It is possible that in our model, ACE1 could not degrade Aβ_42_ due to other competing amyloid-degrading proteases expressed in SH-SY5Y cells or steric hinderance when accessing the active site (40). While clinical studies have proposed that ACE1 may protect the AD brain by degrading Aβ_42_ to Aβ_40_, our data challenge this assumption. These results suggest that mutant ACE1 may not consistently contribute to amyloid clearance and, under certain conditions, could shift the enzyme’s role toward promoting other pathogenic peptides, such as Ang II.

These results align with our previous report characterizing the rare AD-associated R1279Q (R1284Q murine) mutation *in vivo*, which induced hippocampal neurodegeneration linked to overproduction of Ang II and increased activation of the central RAS (26). Unlike the T916M and N1036K mutations, the R1279Q mutation caused an increase in ACE1 in neurons, which accounted for the increased production of Ang II, rather than the mutation altering the catalytic properties of the enzyme (Fig. 4). We and others have shown that increased activation of AT1R by Ang II can cause neuron stress, gliosis and can lead to neurodegeneration (26, 41, 42, 43, 44). However, it is possible that other ACE1 substrates could influence neuronal or glial stress mechanisms in disease, such as bradykinin, Ac-SDKP, substance P, and complement C3, which should be further investigated (12). Further research is required to better understand the potential neurodegenerative mechanisms involving the central RAS.

An important functional characteristic of ACE1 is its presence at the cell membrane, and the ectodomain can be shed into the extracellular matrix. We investigated ACE1 abundance at the cell membrane using flow cytometry on non-permeabilized cells (Fig. 2). This experiment confirmed our previous report that the R1279Q mutation has increased ACE1 at the cell surface and provided new data showing that the T916M mutation had reduced ACE1 at the cell surface compared to controls. Notably, rs3730043 on a separate *ACE* isoform corresponds to an amino acid change at T342M, which results in nonsense-mediated decay of ACE1. Although we did not identify any evidence that this occurred in our T916M mutant line, reduced ACE1 expression in neurons could be a mechanism to counterbalance higher catalytic activity in the brain. This mutation is not located near the stalk region where the enzyme is cleaved to release soluble ACE1, however it has been shown that ACE1 domain point mutations can cause conformational changes which allow for easier cleavage of the stalk region (45, 46, 47). Notably, additional experiments showed that T916M is more stable in our cell lines compared to Wild-type, which may be a pathogenic mechanism of ACE1 in aged neurons (Fig. S3).

This study has limitations that should be considered. Firstly, *ACE* variants, which were discovered in human genome datasets, are very rare and therefore do not represent the majority of sporadic AD cases. Additionally, while our stable cell lines provided a useful tool for experimental modulation of protein expression and function, we cannot rule out the potential effects of ACE1 protein overexpression. Finally, as discussed above, our *in vitro* model system does not recapitulate the cellular architecture or diversity of the brain, so careful consideration is required when interpreting these results.

Overall, we have discovered novel rare coding variants in *ACE* that are associated with LOAD families and characterized them in a cellular model system. T916M and N1036K ACE1 mutations increased the enzyme activity of ACE1, leading to an increase in Ang II production. These results provide further evidence for the role of ACE1 in AD as well as the rationale for continuing research on the central RAS to better understand related mechanisms that may contribute to AD pathogenesis.

## Experimental procedures

### Genetic analyses

We have used two WGS datasets and one WES dataset with AD cases and controls from the National Institute of Mental Health (NIMH) and the National Institute of Aging Alzheimer’s Disease Sequencing Project (NIA ADSP, Table S1). Sequencing and quality control in the NIMH cohort were previously described (48, 49). Sequencing data for the NIA ADSP cohort were obtained from the National Institute on Aging Genetics of Alzheimer’s Disease Data Storage Site (NIAGADS) under accession number: NG00067.v9. There was a small subset of subjects that were collected under a family-based design. Those subjects are described as NIA ADSP families (Table S1). The unrelated NIA ADSP cohort was divided into four subpopulations based on self-reported race and ethnicity: non-Hispanic whites (NHW), African-Americans (AA), Hispanic subpopulation (HISP), and Asian subpopulation. The full list of statistical data for each variant and cohort is provided in Table S2. We have verified the population assignment based on PCA using the Jaccard matrix and removed outliers that were more than five standard deviations away from the mean based on each of the first 10 PCs.GEMINI and SNPEff tools were used to perform functional annotation of the variants. In family-based datasets, we used the Family-Based Association Test (FBAT) Toolkit (https://sites.google.com/view/fbat-web-page) to perform association analysis. We used an offset of 0.15, when modeling the phenotype in FBAT. Given the low minor allele frequency and hence the low number of informative families, we did not have enough statistical power for most rare variants. For case-control datasets, we used PLINK to perform logistic regression with covariates. R statistical software was used for any additional analyses. Pedigree charts were created using Progeny Genetics Pedigree Builder (https://pedigree.progenygenetics.com/).

### Creation and maintenance of SH-SY5Y stable cell lines

Point mutations were added to the Mammalian Expression Vector produced by VectorBuilder containing either Wild-type, mutated (T916M, N1036K, R1279Q), or no *ACE* ORF (Vector) under the CMV promoter and including a neomycin selection cassette. P3 SH-SY5Y neuroblastoma cells (ATCC CRL-2266) were transfected with expression plasmids using lipofectamine 2000 (Thermo Fisher 11,668,027) in media not containing antibiotics. After 6 h, plates were changed to Complete Media [DMEM (Gibco 11,995,065), 10% Fetal Bovine Serum (Gibco A5670701), 1% Penicillin/Streptomycin (Gibco, 15,140,122)]. After 2 days of expression, cells were split into three 10 cm plates in Selection Media 1 [DMEM, 10% FBS, 1% Pen/Strep, 500 μg/ml Geneticin (Gibco, 10,131,035)]. Media changes were made every 2 days for 1 week. Selection Media 2 (DMEM, 10% FBS, 1% Pen/Strep, 200 μg G418) was used for media changes every 3 to 4 days thereafter until G418-resistant colonies were present. 20 colonies per plasmid were picked and replated in 12-well dishes in Maintenance Media (DMEM, 10% FBS, 1% Pen/Strep, 100 μg G418). Colonies were grown to confluency and then were collected to test for ACE1 protein expression *via* Western blot. Clones expressing detectable ACE1 protein were expanded; one viable clone per plasmid was chosen for experimentation. For all experiments, stable cell lines were plated in Maintenance Media containing 10 μM retinoic acid (Sigma-Aldrich R2625) and allowed to differentiate for 5 days. A media change was made on Day 3 of differentiation. Unless otherwise noted, cell lines were plated and assayed in triplicates and experiments were repeated with two or three separate differentiations.

### Immunofluorescence, confocal microscopy, and TUNEL assay

150,000 cells were plated on 18 mm coverglass (Warner Instruments 640,714). After 5 days, cells were gently washed with 1X phosphate-buffered saline (PBS), then incubated in 4% paraformaldehyde (PFA) at 37 °C for 15 min. After being gently washed three times with 1X PBS, cells were placed in blocking/permeabilization buffer (0.1% saponin, 5% donkey serum, 1XPBS) for 1 h, washed, and then incubated with primary antibodies (Table S2) overnight at 4C in blocking/permeabilization buffer. The next day, cells were washed and incubated with secondary antibodies for 2 h in blocking/permeabilization buffer. After being washed, cover glass was mounted on slides using ProLong Gold Antifade Mountant (Thermo Fisher P36930). Z-stack images (5 μm thickness) of ACE1-stained cells were taken on the Nikon AXR confocal microscope using the same acquisition settings for each coverglass. Max projections were created using ImageJ. For TUNEL assay, manufacturer protocol was followed (*In Situ* Cell Death Detection Kit, TMR red, Roche 12,156,792,910); a 30 min incubation with DNase I (1000 U/ml in 50 mM Tris-HCl, pH 7.5, 10 mM MgCl2, 1 mg/ml BSA) was used as a positive control. Three images per coverglass were acquired using the Nikon Ti2 Widefield. To quantify TUNEL-positive DAPI nuclei, NDS files were loaded into ImageJ software, DAPI and TUNEL color channels were split and then converted into 8 bit images. A Gaussian blur filter (1.0 scaled units) was used on each image. After thresholding, binary images were processed using the Watershed tool to split nuclei that had been merged during thresholding. Nuclei were counted using Analyze Particles, and the number of TUNEL nuclei was divided by the total number of nuclei (stained with DAPI).

### Protein extraction, Western blotting, and ACE1 quantification

300,000 cells were plated in 12-well plates. On day 5, media were collected, and cells were lysed with 3-[(3-cholamidopropyl)dimethylammonio]-1-propanesulfonate (CHAPS) lysis buffer (1% CHAPS, 20 mM HEPES, 50 mM Tris-HCl pH 7.4, 100 mM NaCl, 5 mM EDTA, 5 mM EGTA, pH 7.5) on ice. Media was centrifuged at 18,000*g* for 15 min to remove debris, and the supernatant was moved to fresh tubes before storing at −80 °C. Lysate was placed on ice for 45 min before being centrifuged at 18,000*g* for 12 min and the supernatant was moved to fresh tubes before storing at −80 °C. Protein concentration was determined using Pierce BCA Protein Assay. Western blotting samples were prepared containing 1X LDS Sample Buffer (Invitrogen NP0008) and 4% β-mercaptoethanol (Sigma-Aldrich M6250). 5-10 μg of each sample was separated into a 4 to 12% NuPAGE Bis-Tris protein gel (Invitrogen WG1402BOX). Protein was transferred onto nitrocellulose membranes using the Trans-Blot Turbo Transfer System (Bio-Rad). Western blots for ACE1 quantification were transferred overnight (∼16 h) at 20V to polyvinylidene difluoride (PVDF) membranes. Membranes were washed in wash buffer (1X PBS + 0.1% Tween20) and blocked with SuperBlock Blocking Buffer (Invitrogen 37,515). Membranes were incubated in antibody buffer (wash buffer containing 10% SuperBlock) with primary antibodies (Table S2) overnight at 4 °C, washed, and then incubated in antibody buffer with secondary antibodies for 1 h. After washing, signals were developed using SuperSignal West Femto (Invitrogen 34,094) or SuperSignal West PICO Plus (Invitrogen 34,580) and imaged on the ChemiDoc MP Imaging System (Bio-Rad). Signals were analyzed using Image Lab software (Bio-Rad). ACE1 was quantified in lysate samples using ELISA (Abcam, ab263889) for all experiments except those in Figure S2.

### Transient transfection of HEK293T cells

300,000 HEK293T cells (ATCC CRL-3216) were plated in 12-well plates. Lipofectamine 2000 (Thermo Fisher 11,668,027) was used to transfect cells with Wild-type, mutant, or empty backbone plasmids (1.6 μg per well) in media not containing antibiotics. After 6 h, the media was replaced with Complete Media. The plasmids were allowed to express for 24 h before cells were lysed in 1% CHAPS buffer as described above. Samples were prepared for Western blotting and assayed as described above.

### Cycloheximide treatment

300,000 cells were plated in 12-well plates and differentiated for 5 days in retinoic acid. On day 5, cells were treated with 50 μg/ml cycloheximide. Whole cell extracts were collected in 1% CHAPS buffer at 0, 8, 24, and 32 h and processed for western blotting as described above. 5 μg of protein was run for each sample, and blots were probed for ACE1 and β-actin. ACE1 was quantified and normalized to β-actin for each sample. Percentage of ACE1 at 0 h was calculated for each sample at each timepoint, then plotted over time before a 2-way ANOVA was performed to compare the percentage of ACE1 remaining of each cell line to Wild-type (see details in Fig. S3 and Table S1).

### Flow cytometry

300,000 cells were plated in 12-well plates. On day 5, cells were trypsinized and each well was split into three wells of a 96-well plate; all steps thereafter were performed on ice. Cell suspensions were washed in 1X PBS before incubating in 1:1000 Live/Dead fixable dye and 1:200 Human Fc block for 15 min in FACS buffer (2% FBS in 1X PBS). After further washing in 1X PBS, cells were incubated with 1:1000 Anti-human CD153 for 15 min in FACS buffer and then fixed in 2% PFA for 15 min. After washing, cells were resuspended in 50 μL of FACS buffer and surface ACE1 expression was collected on BD FACSymphony (BD Bioscience), gated for live cells (Fig. S5), and analyzed using FlowJo.

### ACE1 activity assay

For ACE1 activity assays, 300,000 cells were plated in 12-well plates. On day 5, media was changed to Opti-MEM and cells were lysed with manufacturer’s Lysis Buffer (Abcam ab239703) on ice after 24 h. Samples were processed as described above and were stored at −80 °C if not assayed on the same day as collection. Samples were prepared according to the manufacturer’s protocol, including at least one sample per cell line which was treated with 15 μM Captopril. ACE1 activity was measured kinetically for 3 h (Abcam ab239703). Area under the curve was calculated for each sample and was normalized to ACE1 protein (Abcam ab263889) in each reaction.

### Ang II and Aβ_42_ treatments

For Ang II measurements, 300,000 cells were plated in 12-well plates. On day 5, one well per cell line was pre-treated for 30 min with 15 μM captopril (Sigma-Aldrich C4042). Media was then changed to Opti-MEM containing 500 nM of Ang I (Sigma-Aldrich A9650), 500 nM of Ang I with 15 μM captopril, or vehicle. After 1 h, the media was collected on ice, centrifuged at 18,000*g* for 15 min, and then assayed for Ang II by ELISA (Sigma-Aldrich RAB0010). Lysate was collected in 1% CHAPS buffer, centrifuged at 18,000*g* after resting on ice for 45 min, and then stored at −80 °C. Ang II measurements were normalized to ACE1 protein in the lysate (Abcam, ab263889).

For Aβ_42_ treatments, 300,000 cells were plated in 12-well plates. On day 5, Aβ_42_ HFIP (rPeptide A-1163–2) was prepared as described previously (50). Cells were treated with 1 nM of Aβ_42_ oligomers or vehicle (DMSO prepared the same as Aβ_42_ oligomers) in Opti-MEM (“Sample Aβ_42_”). 1 nM of Aβ_42_ oligomers was added to a well with no cells to act as a control and for analysis calculations (“1 nM control”, see below). After 24 h, the media was collected and placed on ice and the lysate was collected as described above. The media was diluted 1:10 to be assayed for Aβ_42_ *via* ELISA (Thermo Fisher KHB3441). The difference between the 1 nM control and the Sample Aβ42 was calculated to determine how much Aβ_42_ was lost (through degradation or other means) during the treatment. Media was assayed for Aβ_40_ *via* ELISA (Thermo Fisher KHB3481).

### Cell-less in vitro Ang I and Aβ_42_ incubations with sACE1

To prepare sACE1, 300,000 cells were plated in 12-well plates and differentiated for 5 days. On day 5, conditioned media was collected on ice and assayed for ACE1 protein (abcam ab263889). Reactions were performed in sterile 96-well plates at 37 °C. For Ang I reactions, 3 ng of sACE1 was added to a well with 100 nM Ang I or vehicle with or without 15 μM Captopril and placed at 37 °C for 1 h. Reactions were then diluted 1:10 to be assayed for Ang II *via* ELISA (Sigma-Aldrich RAB0010). For Aβ_42_ reactions, 3 ng of sACE1 was added to a well with 1 nM Aβ_42_ oligomers (prepared as described above) or vehicle with or without 15 μM Captopril and placed at 37 °C for 24 h 1 nM of Aβ_42_ oligomers was added to a well with no cells to act as a control and for analysis calculations. Reactions were then diluted 1:10 to be assayed for Aβ_42_ *via* ELISA (Thermo Fisher KHB3442). The amount of Aβ_42_ that was lost (through degradation or other means) during the treatment was calculated as described previously.

### Using AlphaFold3 to create predictive mutant ACE1 structures

Point mutations T916M and N1036K were introduced into the amino acid sequence of ACE1 C-domain (4APH, Protein Data Bank) which represents amino acids 642 to 1230 of full-length ACE1 P12821 (ACE_HUMAN, Uniprot) (51). Sequences were submitted to AlphaFold Server (AlphaFold3) (52). Mutated structures were aligned to 4APH using PyMOL (The PyMOL Molecular Graphics System, Version 3.0 Schrödinger, LLC.). Measurements were made between Thr75-Val148 and Pro163-Thr311, distances for 4APH (Wild-type), T916M, and N1036K are reported in Figure S5.

### Statistical analysis

Statistics were performed using GraphPad. Unless otherwise noted in figure legend, One-way ANOVA was first performed with Brown-Forsythe and Bartlett’s test for different standard deviations (SDs). If SDs were significantly different (*p* < 0.05), Brown-Forsythe ANOVA test was performed with Dunnett's T3 multiple comparisons test (comparing all columns to WT). If SDs were not significantly different, One-way ANOVA was performed with Dunnett's multiple comparisons test (comparing all columns to WT). All individual data points and statistical test details are provided in Data Set 1. ∗ = *p* < 0.05, ∗∗ = *p* < 0.01, ∗∗∗ = *p* < 0.001, ∗∗∗∗ = *p* < 0.0001.

## Data availability

All data associated with this study are present in the paper or the supporting information.

## Supporting information

This article contains supporting information.

## Conflict of interest

The authors declare that they do not have any conflicts of interest with the content of this article.
